# Epidemiology of temporomandibular joint disorders and related painful conditions

**DOI:** 10.1186/1744-8069-10-S1-O16

**Published:** 2014-12-15

**Authors:** Gary D Slade

**Affiliations:** 1 Center for Pain Research and Innovation, University of North Carolina at Chapel Hill, Chapel Hill, NC 27599, USA

## 

Epidemiology uncovers patterns of disease distribution in human populations and seeks determinants of those patterns. With recent emphasis on chronic pain as "a disease in itself", it is informative to compare how the *distribution* of temporomandibular joint disorders (TMJD) compares with that of related pain conditions. Over two decades of National Health Interview Surveys (1989 to 2009), the prevalence of self-reported TMJD symptoms remained stable, affecting 5% of U.S. adults. In 2009, prevalence was greater in females than males, and increased with age to midlife before decreasing in older age. While racial- and ethnic-group differences were small, there was a pronounced income gradient, with greater prevalence at lower household income. Similar distributions according to gender, age and income occurred for headache and neck pain, although not for low back pain. There was also marked overlap of TMJD with those related pain conditions, irrespective of whether they occurred above or below the shoulders. Moreover, there was significant overlap of TMJD with non-painful medical conditions. In order to understand reasons for this overlap, prospective studies of TMJD incidence are needed to discover *determinants* of the disease. In the community-based OPPERA prospective cohort study, TMJD incidence was measured in 2,737 adults aged 18-44 years who had no significant history of TMJD when enrolled. During three years of follow-up, 19% of people per annum developed TMD symptoms and for a quarter of symptomatic episodes, pain intensity was severe. Examiner-verified, first-onset TMJD developed at an annual rate of 3.5% per annum, although the rate was approximately doubled in study participants who, at enrollment, reported related pain conditions. Likewise, TMJD incidence was strongly associated with a checklist of 20 non-specific health conditions reported at enrollment, ranging from depression to sleep apnea. Yet, by virtue of the study design, study participants had no TMJD at enrollment, meaning that the related pain conditions and other health conditions did not "overlap" concurrently with TMJD. Instead, they represent risk factors for development of TMJD. In fact, in multivariable analysis, related pain and other health conditions were among the strongest predictors of first-onset TMD. Furthermore, their effects on risk of developing TMJD were independent of conventional risk factors for TMJD.

## Conclusion

Impaired general health, whether painful or not, is an important risk factor for development of painful TMJD.

## Disclosures

Gary Slade is a consultant and shareholder in Algynomics Inc., a company that does pain research.

